# Effectiveness of Telehealth Interventions for Women With Postpartum Depression: Systematic Review and Meta-analysis

**DOI:** 10.2196/32544

**Published:** 2021-10-07

**Authors:** Liuhong Zhao, Jingfen Chen, Liuying Lan, Ni Deng, Yan Liao, Liqun Yue, Innie Chen, Shi Wu Wen, Ri-hua Xie

**Affiliations:** 1 Department of Nursing The Seventh Affiliated Hospital Southern Medical University Foshan China; 2 Department of Obstetrics and Gynecology The Seventh Affiliated Hospital Southern Medical University Foshan China; 3 Clinical Epidemiology Program Ottawa Hospital Research Institute Ottawa, ON Canada; 4 Department of Nursing The Affiliated Hospital of Guangdong Medical University Zhanjiang China; 5 Department of Obstetrics & Gynecology Faculty of Medicine University of Ottawa Ottawa, ON Canada; 6 School of Epidemiology and Public Health Faculty of Medicine University of Ottawa Ottawa, ON Canada

**Keywords:** telehealth, postpartum depression, anxiety, meta-analysis

## Abstract

**Background:**

Postpartum depression (PPD) is a prevalent mental health problem with serious adverse consequences for affected women and their infants. Clinical trials have found that telehealth interventions for women with PPD result in increased accessibility and improved treatment effectiveness. However, no comprehensive synthesis of evidence from clinical trials by systematic review has been conducted.

**Objective:**

The aim of this study is to evaluate the effectiveness of telehealth interventions in reducing depressive symptoms and anxiety in women with PPD. To enhance the homogeneity and interpretability of the findings, this systematic review focuses on PPD measured by the Edinburgh Postnatal Depression Scale (EPDS).

**Methods:**

PubMed, The Cochrane Library, CINAHL, PsycINFO, CNKI, and Wanfang were electronically searched to identify randomized controlled trials (RCTs) on the effectiveness of telehealth interventions for women with PPD from inception to February 28, 2021. Data extraction and quality assessment were performed independently by two researchers. The quality of included studies was assessed using the Cochrane risk-of-bias tool, and meta-analysis was performed using RevMan 5.4 software.

**Results:**

Following the search, 9 RCTs with a total of 1958 women with PPD were included. The EPDS (mean difference=–2.99, 95% CI –4.52 to –1.46; *P*<.001) and anxiety (standardized mean difference=–0.39, 95% CI –0.67 to –0.12; *P*=.005) scores were significantly lower in the telehealth group compared with the control group. Significant subgroup differences were found in depressive symptoms according to the severity of PPD, telehealth technology, specific therapy, and follow-up time (*P*<.001).

**Conclusions:**

Telehealth interventions could effectively reduce the symptoms of depression and anxiety in women with PPD. However, better designed and more rigorous large-scale RCTs targeting specific therapies are needed to further explore the potential of telehealth interventions for PPD.

**Trial Registration:**

PROSPERO CRD42021258541; https://www.crd.york.ac.uk/prospero/display_record.php?RecordID=258541

## Introduction

Postpartum depression (PPD) is one of the most common mental health disorders in women after giving birth. A systematic review comprised of 58 articles with a total of 37,294 women reported that the overall prevalence of PPD was 17% among healthy mothers [1]. PPD symptoms in women manifest as sleep disorders, mood swings, sadness and crying, loss of appetite, a lack of interest in daily activities, or even more serious adverse outcomes such as suicide and infanticide [2]. PPD may also be associated with an increased risk of cognitive and behavioral problems in infants [3]. Timely access to effective interventions such as psychotherapy and pharmacotherapy is important for women affected by PPD [4,5]. However, breastfeeding mothers may have concerns regarding their infant’s exposure to medications because of reported side effects of antidepressant exposure in infants (eg, excessive crying, colic, irritability, sedation, poor feeding, and sleep problems) [6]. Therefore, antidepressant medications are recommended only for women with severe depression, while psychotherapy is the first-line method for prevention and treatment of mild to moderate PPD [7]. The clinical effectiveness of common psychotherapies for PPD such as peer support therapy [8], interpersonal therapy [9], cognitive behavioral therapy [10], and mindfulness therapy [11] has been demonstrated. However, psychotherapy conducted in a traditional face-to-face manner may not be accessible for many women due to time and financial hurdles, childcare concerns, and fear of social stigma [12].

Women who have challenges accessing face-to-face psychotherapy may benefit from telehealth interventions, through which health care and health education could be provided to them at home [13]. Telehealth technologies include telephones, websites, videoconferences, and apps that allow consultation, assessment, and intervention services to be provided remotely by health professionals or peer support [14], which have been widely used to help manage diseases in various domains, including diabetes self-management [15], pulmonary rehabilitation [16], and palliative home care [17]. In addition, telehealth has been gaining momentum in continuous obstetrical care [18]. Telehealth facilitates interactions and communication between specialists and patients in the field of maternal-fetal medicine, especially in the postpartum period for breastfeeding and lactation assistance in rural communities [19]. Telehealth care has many benefits, including increased access and convenience and decreased social stigma and costs [20].

A previous study [21] showed that there are 18 scales for screening depression symptoms, including the Patient Health Questionnaire-9 Item [22], the Beck Depression Inventory II scale [23], the Postpartum Depression Screening Scale [24], and the Edinburgh Postnatal Depression Scale (EPDS) [25,26]. These scales have different sensitivity, specificity, and disease predictivity. Of them, EPDS is the most reliable scale in terms of disease predictivity and adaptivity for differences in population profiles, and it has therefore been the most frequently used scale in clinical and research settings to screen for PPD [25,26]. To enhance the homogeneity and interpretability of the findings, this systematic review targets adult women with PPD measured by EPDS [25,26].

## Methods

### Overview

This systematic review was conducted following the guidelines of the PRISMA (Preferred Reporting Items for Systematic Reviews and Meta-Analyses) statement [27]. The study protocol was registered in PROSPERO (the International Prospective Register of Systematic Reviews) as CRD42021258541.

### Search Strategy

For this study, 4 English databases (PubMed, the Cochrane Library, CINAHL, and PsycINFO) and 2 Chinese databases (CNKI and Wanfang) were electronically searched to identify randomized controlled trials (RCTs) on the effectiveness of telehealth interventions for women with PPD from inception to February 28, 2021. Advanced searches were performed using a combination of two groups of terms according to the syntax rules of each database: (1) telehealth-related terms, including telehealth, telemedicine, telecommunication, telephone, remote consultation, information technology, mobile health, m-Health, e-Health, internet, web-based, social media, application, and software, and (2) PPD-related terms, including postpartum depression, PPD, post-partum depression, postnatal depression, post-natal depression, maternal depression, postpartum mental disorder, and puerperal disorder. In addition, ClinicalTrials.gov, the World Health Organization International Clinical Trials Registry Platform, and the Chinese Clinical Trial Register were searched for unpublished trials relevant to this review.

### Inclusion and Exclusion Criteria

To be eligible, RCTs had to meet the following criteria: (1) target adult women with EPDS scores ≥9 points; (2) use telehealth interventions including mobile phones, apps, websites, or other remote technologies compared with routine care in the control group (participants in the control group were inaccessible to any telehealth technologies, but were free to receive routine care including any offline treatment at public hospitals or maternal and child health care centers); (3) assess the primary outcome of depression symptoms using EPDS, and secondary outcomes including any improvement of social support, loneliness, and anxiety using any scale; and (4) be published in English or in Chinese.

Studies were excluded for the following reasons: (1) study included women with severe physical illnesses, a history of mental illnesses and medication treatments, drug and/or alcohol abuse, or suicidal tendency; (2) study included women whose infants had adverse neonatal outcomes such as 5-minute Apgar score <5 points, assisted ventilation for more than 6 hours, neonatal seizure, birth injury, or neonatal death; and (3) study was an RCT protocol or duplicate.

### Study Selection

The software Endnote X9 (Clarivate Analytics) was used to import all the references and remove duplicates. The remaining studies were assessed against the inclusion and exclusion criteria by two independent reviewers (LZ and JC). Study selection was conducted in a stepwise manner. First, titles and abstracts of all studies were independently screened for potential eligibility. Any disagreements were discussed until consensus was reached. Second, the full papers of all included abstracts were independently assessed. Any discrepancies that arose during the assessment were resolved by a third reviewer (RHX).

### Quality Assessment

The risk of bias was assessed according to the guidelines provided by the Cochrane risk-of-bias tool for randomized trials (version 2.0) [28]. Risk ratings of “low risk,” “unclear,” and “high risk” were assigned to each type of bias based on the presence of selection bias, performance bias, detection bias, attrition bias, reporting bias, and other bias. Any disagreements with respect to study quality were resolved by a third reviewer (RHX).

### Data Extraction

The data were extracted independently by two reviewers (LZ and JC) at the same time and disagreements were resolved by consensus. Data extracted from each relevant trial included author, the year of publication, country, participant, inclusion criterion (EPDS scores), sample size, telehealth technology, specific therapy, follow-up time, and outcomes (primary and secondary outcomes) with measure scales. Any disagreements between the two reviewers were resolved by a third reviewer (RHX).

### Data Synthesis and Analysis

RevMan 5.4 software (The Cochrane Collaboration) was applied in the meta-analysis of the data. The effect estimate was expressed as means and standard deviations (SD) for continuous data. The standardized mean differences (SMD) with their corresponding 95% CI were applied to combine studies that measured the same outcome with different scales. If the same scales were used to evaluate one outcome, mean difference (MD) with its 95% CI could be employed. MD was derived from inverse variance methods. If the SD was not reported, it was computed from standard error (SE) following the Cochrane Handbook [28]: 
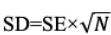
. Heterogeneity was assessed by *I²*, with significant statistical heterogeneity as *I²*>50%. The fixed-effect model was used to estimate one true effect in cases without significant heterogeneity (*I^2^*<50%), whereas the random-effect model was employed to estimate the effects in cases with significant heterogeneity between studies (*I²*>50%). When there was significant heterogeneity, data would be double-checked and then subgroup analysis or sensitivity analysis was performed to explore the sources of heterogeneity. A *P* value <.05 was considered statistically significant.

## Results

### Search Results

**Figure 1:**
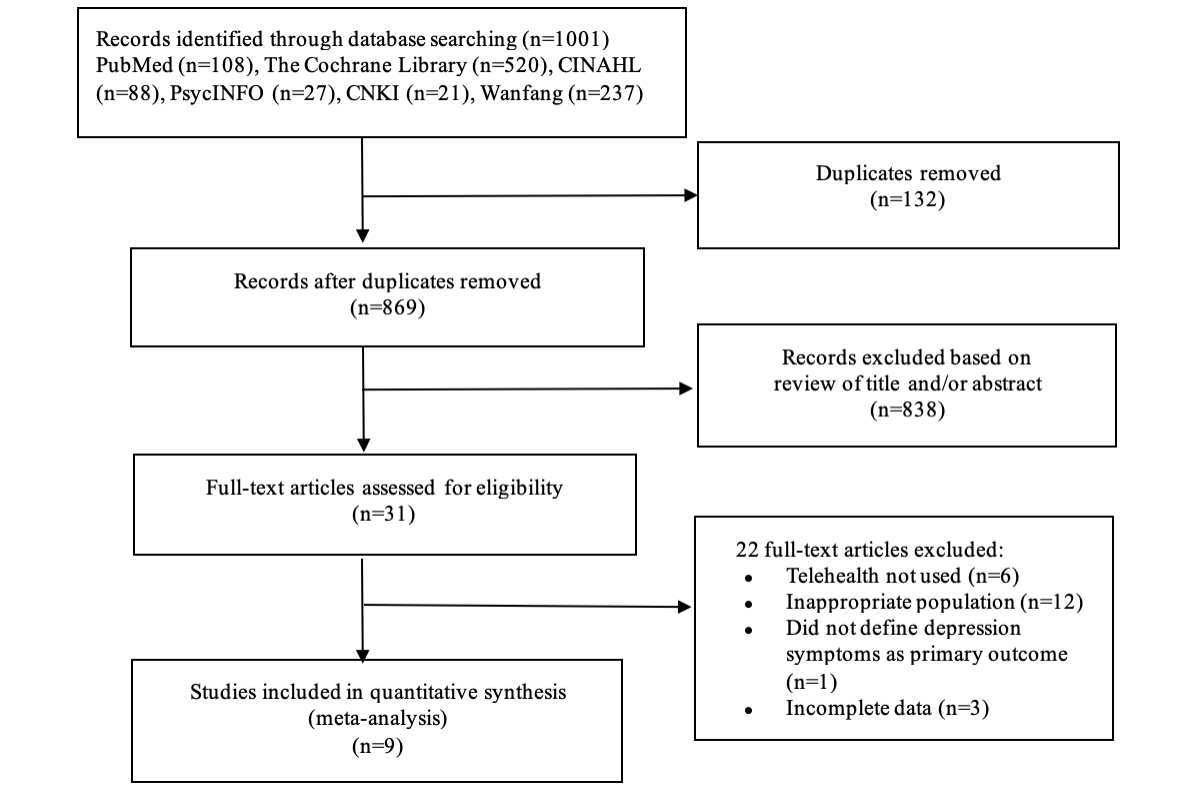
Flow diagram of study selection.

The PRISMA flow diagram for this study is shown in Figure 1. The search strategies yielded 1001 potentially relevant citations from the 6 electronic databases searched. After excluding duplications and screening titles and abstracts for eligibility, 31 studies were retained for full-text evaluation. Of them, 22 studies were excluded according to the inclusion and exclusion criteria. A total of 9 studies [29-37] were included in this systematic review.

### Characteristics of Included Studies

This systematic review included 9 RCTs [29-37] with a total of 1958 participants. The 9 studies included were conducted in Singapore, Canada, Portugal, Iran, China, and the United Kingdom and were published between 2003 and 2020. Participants were postpartum women aged ≥18 years. All recruited participants had baseline EPDS scores ≥9 points. The sample sizes ranged from 42 to 910. The 9 included studies applied telehealth technologies including telephones [29-31,34,36,37], apps [33,37], and websites [32,35,36]. Of them, 3 studies [29,30,37] used peer support therapy, 5 studies [32-36] used cognitive behavioral therapy and behavioral activation therapy, and 1 study [31] used interpersonal therapy. The follow-up period of these 9 studies [29-37] ranged from 4 weeks to 36 weeks after completion of telehealth interventions. Characteristics of the 9 studies included are presented in Multimedia Appendix 1.

### Risk of Bias

**Figure 2:**
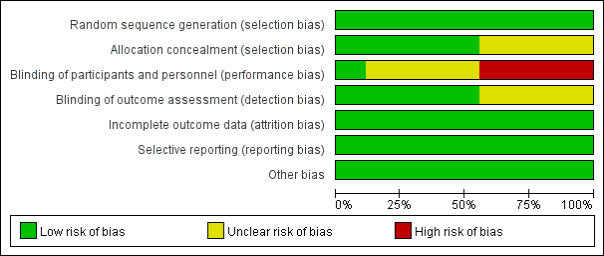
Risk of bias graph.

We summarized the findings for risk of bias in Figure 2 and Multimedia Appendix 2. Among the 9 RCTs [29-37], 5 studies [30-33,37] used a computer-generated random sequence, 1 study [34] used a random number table, 2 studies [35,36] used a minimization algorithm including a stochastic element, and 1 study [29] only mentioned the word “random,” with no details of the randomization method used. Allocation concealment was done in 5 studies [29,34-37]. Furthermore, 1 study [37] implemented the blinding of participants and personnel and outcome assessment, 4 studies [32,33,35,36] were unclear about the blinding of outcome assessment, and 4 studies [30,31,33,34] had high risk of bias on implementing the blinding of participants and personnel. All 9 studies included had low risk of bias on incomplete outcome data, selective reporting, or other bias.

### Meta-Analysis

#### Depressive Symptoms in the Telehealth Group and the Control Group

**Figure 3:**
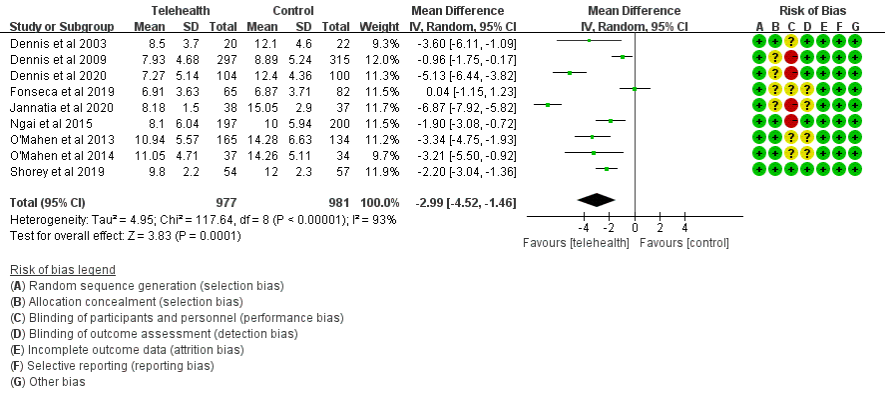
Depressive symptom scores in the telehealth group and the control group.

The 9 included studies [29-37] assessed the effectiveness of telehealth interventions on depressive symptoms among 1958 women by comparing the telehealth group and the control group. Depressive symptoms were all measured using EPDS. Considerable heterogeneity among studies was detected (*I^2^*>50%); thus, a randomized model was used. The overall pooled analysis demonstrated that total EPDS scores of women with PPD who received telehealth interventions were significantly lower than the control group (MD=–2.99, 95% CI –4.52 to –1.46; *P*<.001; *I^2^*=93%; Figure 3).

#### Social Support in the Telehealth Group and the Control Group

**Figure 4:**
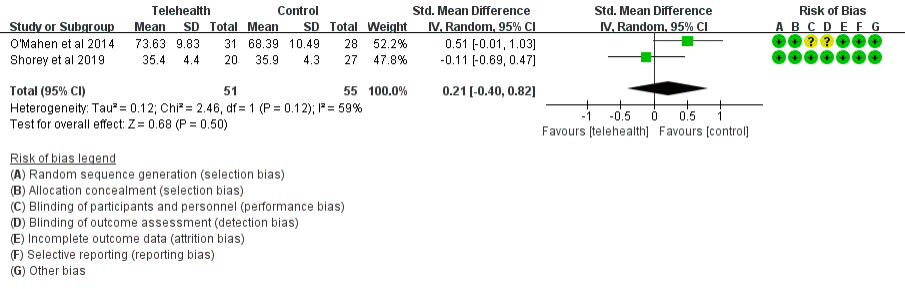
Social support scores in the telehealth group and the control group.

A total of 2 studies [36,37] reported social support improvement after interventions by the Perceived Social Support for Parenting instrument [37] and the Social Provision Scale [36]. The efficacy of telehealth was evaluated among 101 women by comparing the telehealth and control groups. There was no statistically significant difference between the two groups (SMD=–0.21, 95% CI –0.40 to 0.82; *P*=.50, *I^2^*=59%; Figure 4).

#### Loneliness in the Telehealth Group and the Control Group

**Figure 5:**
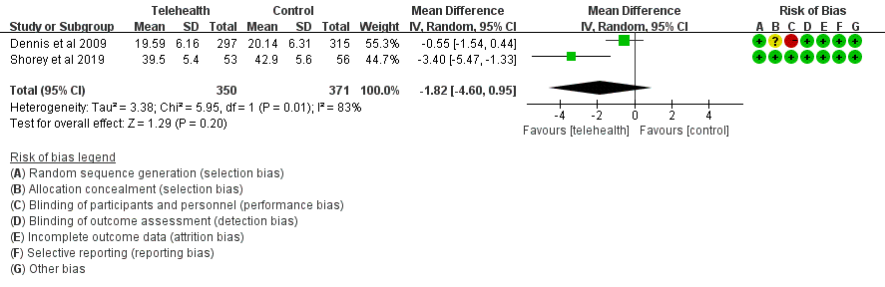
Loneliness scores in the telehealth group and the control group.

A total of 2 studies [30,37] reported loneliness scores after interventions using the University of California, Los Angeles Loneliness Scale. The efficacy of telehealth was evaluated among 721 participants by comparing the telehealth and control groups. Meta-analysis showed no statistically significant difference between the two groups (MD=–1.82, 95% CI –4.60 to 0.95; *P*=.20, *I^2^* = 83%; Figure 5).

#### Anxiety in the Telehealth Group and the Control Group

**Figure 6:**
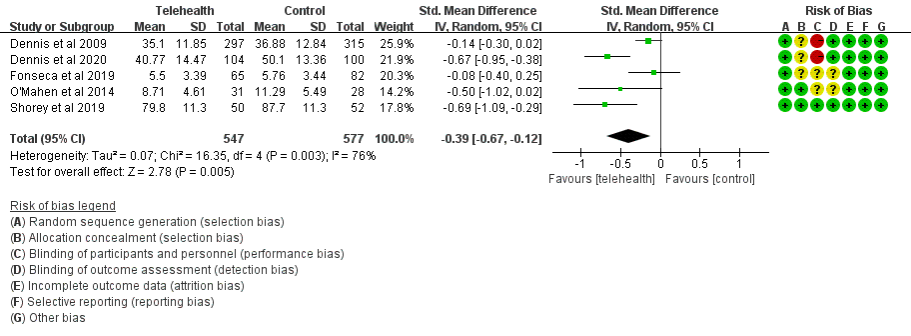
Anxiety symptom scores in the telehealth group and the control group.

A total of 5 studies [30-32,36,37] presented anxiety symptoms after interventions. Anxiety symptoms were measured through the State-Trait Anxiety Inventory [30,31,37], the Hospital Anxiety and Depression Scale [32], and the Generalized Anxiety Disorder 7-item scale [36]. Scores of anxiety symptoms in the telehealth group were lower than in the control group (SMD=–0.39, 95% CI –0.67 to –0.12; *P*=.005; *I^2^*=76%; Figure 6).

### Sensitivity and Subgroup Analysis

In relation to the primary outcome (ie, depressive symptoms improvement), the pooled estimates were consistent when excluding one study at a time, indicating that the meta-analysis results of this study were stable and reliable. A series of subgroup analyses were performed to uncover more information about the heterogeneity (as shown in Table 1). Subgroup analyses were performed according to the severity of PPD, telehealth technology, specific therapy, and follow-up time. However, considerable heterogeneity (*I^2^*≥71%) existed in each subgroup analysis.

**Table 1:** Subgroup analyses of the effect of telehealth interventions on depressive symptoms.

Subgroup analyses			Number of randomized controlled trials		Mean difference (95% CI)		*Z* statistic		*P* value		*I*^2^, %
**Severity of postpartum depression**									<.001		
	EPDS^a^ scores ≥9	3 [29,30,37]		–1.64 (–2.20 to –1.08)		5.76		<.001		71	
	EPDS scores ≥10	2 [32,34]		–0.94 (–1.78 to –0.10)		2.2		.03		81	
	EPDS scores ≥12	4 [31,33,35,36]		–5.27 (–5.95 to –4.60)		15.29		<.001		84	
**Telehealth technology**									<.001		
	Telephone	6 [29-31,34,36,37]		–2.18 (–2.64 to –1.72)		9.26		<.001		84	
	App	2 [33,37]		–4.02 (–4.67 to –3.36)		12.03		<.001		98	
	Website	3 [32,35,36]		–1.62 (–2.47 to –0.78)		3.76		<.001		87	
**Specific therapy**									<.001		
	Peer support	3 [29,30,37]		–1.64 (–2.20 to –1.08)		5.76		<.001		71	
	Interpersonal psychotherapy	1 [31]		–5.13 (–6.44 to –3.82)		7.7		<.001		N/A^b^	
	Cognitive behavioral therapy	3 [32-34]		–3.25 (–3.91 to –2.60)		9.73		<.001		97	
	Behavioral activation therapy	2 [35,36]		–3.30 (–4.50 to –2.10)		5.4		<.001		0	
**Follow-up time** **(weeks)**									<.001		
	4	2 [29,37]		–1.21 (–1.93 to –0.49)		3.3		.001		73	
	6	1 [34]		–1.90 (–3.08 to –0.72)		3.16		.002		N/A	
	8	3 [29,32,33]		–3.86 (–4.64 to –3.07)		9.6		<.001		99	
	12	3 [30,31,37]		–2.12 (–2.65 to –1.60)		7.92		<.001		93	
	15	1 [35]		–3.34 (–4.75 to –1.93)		4.65		<.001		N/A	
	17	1 [36]		–3.21 (–5.50 to –0.92)		2.74		.006		N/A	
	24	3 [30,31,34]		–1.58 (–2.14 to –1.03)		5.55		<.001		94	
	36	1 [31]		–3.34 (–4.75 to –1.93)		4.65		<.001		N/A	

^a^EPDS: Edinburgh Postnatal Depression Scale.

^b^N/A: not applicable.

## Discussion

### Overview

Our systematic review summarized the effectiveness of telehealth interventions on PPD and associated maternal mental health problems in the postpartum period (defined as ≤12 months after childbirth). We found that telehealth interventions could significantly improve depression and anxiety symptoms, although their effectiveness in improving social support and reducing loneliness was less certain.

### Effectiveness of Telehealth Interventions on Postpartum Depression

In this study, we demonstrated that maternal depression scores were significantly lower in the telehealth group compared to the control group. Previous studies [30,34,38] were inconsistent regarding whether telehealth could improve maternal depression in the long term. One study [38] reported that the EPDS scores of women in the telehealth group increased at 24 weeks after interventions. Another study [30] discovered no significant difference in EPDS scores at 24 weeks after interventions between the two groups. However, one study [34] reported that EPDS scores at 24 weeks after interventions were significantly lower in the telehealth group. The synthesized result of this systematic review suggested that telehealth was effective in reducing EPDS scores at 24 weeks after the interventions, which was consistent with an earlier systematic review [39] comprised of 7 RCTs with a total of 1106 participants.

### Effectiveness of Telehealth Interventions on Social Support, Loneliness, and Anxiety

In this study, we also summarized the effectiveness of telehealth interventions on mental health issues associated with PPD including social support, loneliness, and anxiety. However, telehealth interventions were not significantly effective at improving social support and loneliness in women affected by PPD. This may be related to the use of different assessment tools and the small number of included studies in the literature reporting on these outcomes. In an internet-based peer therapy project in Singapore [37], the authors found that the degree of loneliness in depressed women was reduced while social support was increased after an online peer support intervention that used telephones and apps. On the other hand, the symptoms of anxiety were significantly reduced in the telehealth group, updating the results in the study which found no evidence on the effectiveness of telehealth for anxiety [40]. Coexistence of PPD and anxiety was common [41] and was mainly related to negative life events experienced by mothers with inadequate social support and increased childcare burden [42]. Results of this systematic review suggested that the potential for telehealth to improve mental health care for either PPD or anxiety is being increasingly recognized by affected women and health care providers [43]. Many women have already used publicly available online apps to access informational support, to consult a team of specialists, or to seek and find resources to alleviate PPD and anxiety [44].

### Strengths and Limitations

This review has multiple strengths. The results of subgroup and sensitivity analyses suggested the findings are robust. More than half of the included trials were of high quality, with a relatively high degree of evidence that telehealth interventions could be effective in PPD treatment. Furthermore, the studies included were conducted in both developed and developing countries, expanding generalizability. Finally, this review analyzed not only the effectiveness of telehealth interventions on PPD, but also on social support, loneliness, and anxiety.

However, there are several limitations in this review. First, there could be selection bias in the original studies as most women participated in the studies on a voluntary basis and were recruited online. Second, most of the included studies relied on self-report measure scales at either recruitment or follow-up, which may lead to inflated estimates of effect sizes. Third, the findings of this meta-analysis were limited by major heterogeneity. There was methodological heterogeneity among the studies included in terms of the severity of PPD, telehealth technology, specific therapy, and follow-up time. In addition, the secondary outcomes in the 9 RCTs included were assessed using different scales.

### Implications for Practice

Through telehealth services, women could have access to the relevant knowledge of psychological interventions anytime and anywhere. The anonymity of chat rooms in telehealth services could help protect women’s privacy, providing a new treatment option for women who do not want to receive a face-to-face treatment due to social stigma.

### Implications for Future Research

This review highlights some directions for future research, including increasing research attention on antenatal and peripartum depression and determining the applicability of telehealth interventions for adolescent mothers who may be more comfortable with novel technologies. Consideration of an intervention including the mother’s partner may be an important approach in future research, especially in cases where triggering events (such as adverse infant outcomes, major negative life events) may affect both the woman and her partner. Future studies should also aim to enhance methodological quality through consistent design and execution on such aspects of the study including telehealth technology, follow-up time, and severity of PPD.

### Conclusion

Telehealth interventions could effectively reduce the symptoms of depression and anxiety in women with PPD. However, better designed and more rigorous large-scale RCTs targeting specific therapies are needed to further explore the potential of telehealth interventions for PPD.

## Appendix

Multimedia Appendix 1 Characteristics of the 9 included randomized controlled trials.

Multimedia Appendix 2 Risk of bias chart.

## Abbreviations

EPDS: Edinburgh Postnatal Depression Scale
MD: mean difference
PPD: postpartum depression
PRISMA: Preferred Reporting Items for Systematic Reviews and Meta-analysis
PROSPERO: International Prospective Register of Systematic Reviews
RCT: randomized controlled trial
SMD: standardized mean difference
