# Supplementary material for: Effectiveness of Telehealth Interventions for Women With Postpartum Depression: Systematic Review and Meta-analysis
Source: JMIR Mhealth Uhealth. 2021 Oct 7;9(10):e32544. doi: 10.2196/32544 (PMC8532017; doi:10.2196/32544)
Supplement: Multimedia Appendix 1 [file mhealth_v9i10e32544_app1.docx]

**Multimedia Appendix 1.** Characteristics of the 9 included RCTs.

| **Author**  **(year)** | **Country** | **Participant** | **Inclusion criterion**  **(EPDS^a^ score)** | **Sample**  **size**  **(T/C^b^)** | **Telehealth technology** | **Specific therapy** | **Follow-up time** | **Outcomes**  **(With measure scales)** |
| --- | --- | --- | --- | --- | --- | --- | --- | --- |
| Dennis et al  (2003) [29] | Canada | 8-12 weeks  postpartum women | > 9 | 20/22 | Telephone | Peer support therapy | 4w, 8w | Depression (EPDS) |
| Dennis et al  (2009) [30] | Canada | within 2 weeks  postpartum women | > 9 | 349/352 | Telephone | Peer support therapy | 12w, 24w | Depression (EPDS)  Anxiety (STAI^c^)  Loneliness (UCLA^d^) |
| Dennis et al  (2020) [31] | Canada | 2-24 weeks  postpartum women | > 12 | 120/121 | Telephone | Interpersonal psychotherapy | 12w, 24w, 36w | Depression (EPDS)  Anxiety (STAI) |
| Fonseca et al  (2019) [32] | Portugal | within 3 months  postpartum women | > 10 | 98/96 | Website | Cognitive behavioral therapy | 8w | Depression (EPDS)  Anxiety (HADS^e^) |
| Jannatia et al  (2020) [33] | Iran | within 6 months  postpartum women | ≥ 13 | 39/39 | App | Cognitive behavioral therapy | 8w | Depression (EPDS) |
| Ngai et al  (2015) [34] | China | within 1 week  postpartum women | ≥ 10 | 197/200 | Telephone | Cognitive behavioral therapy | 6w, 24w | Depression (EPDS) |
| O'Mahen et al  (2013) [35] | The United  Kingdom | within 1 year  postpartum women | > 12 | 462/448 | Website | Behavioral activation therapy | 15w | Depression (EPDS) |
| O'Mahen et al  (2014) [36] | The United  Kingdom | within 1 year  postpartum women | > 12 | 41/42 | Website,  telephone | Behavioral activation therapy | 17w | Depression (EPDS)  Anxiety (GAD-7^f^)  Social Support (SPS^g^) |
| Shorey et al (2019) [37] | Singapore | within 3 months  postpartum women | ≥ 9 | 69/69 | Telephone, app | Peer support therapy | 4w, 12w | Depression (EPDS)  Anxiety (STAI)  Loneliness (UCLA^d^)  Social Support (PSSP^h^) |

References cited in this table: [29-37]

^a^EPDS: Edinburgh Postnatal Depression Scale

^b^T/C: telehealth/control

^c^STAI: State-Trait Anxiety Inventory

^d^UCLA: University of California Los Angeles Loneliness Scale

^e^HADS: Hospital Anxiety and Depression Scale

^f^GAD-7: Generalized Anxiety Disorder 7-item Scale

^g^SPS: Social Provision Scale

^h^PSSP: The Perceived Social Support for Parenting
