# Supplementary material for: Effectiveness of Telehealth Interventions for Women With Postpartum Depression: Systematic Review and Meta-analysis
Source: JMIR Mhealth Uhealth. 2021 Oct 7;9(10):e32544. doi: 10.2196/32544 (PMC8532017; doi:10.2196/32544)
Supplement: Multimedia Appendix 2 [file mhealth_v9i10e32544_app2.docx]

**Multimedia Appendix 2.** Risk of bias chart.

| **Author**  **(year)** | **Random sequence generation**  **(Selection bias)** | **Allocation concealment**  **(Selection bias)** | **Blinding**  **(Performance bias and detection bias)** | | | **Incomplete outcome data**  **(Attrition bias)** | **Selective reporting**  **(Reporting bias)** | **Other bias** |
| --- | --- | --- | --- | --- | --- | --- | --- | --- |
|  |  |  | **participants** | **personnel** | **outcome assessment** |  |  |  |
| Dennis et al  (2003) [29] | No specific method | Seal opaque envelopes | Unclear | Low | Low | Low | Low | Low |
| Dennis et al  (2009) [30] | Computer software | Unclear | High | High | Low | Low | Low | Low |
| Dennis et al  (2020) [31] | Computer software | Unclear | High | High | Low | Low | Low | Low |
| Fonseca et al  (2019) [32] | Computer software | Unclear | Unclear | Unclear | Unclear | Low | Low | Low |
| Jannatia et al  (2020) [33] | Computer software | Unclear | High | Unclear | Unclear | Low | Low | Low |
| Ngai et al  (2015) [34] | Random number table | Seal opaque envelopes | High | Unclear | Low | Low | Low | Low |
| O'Mahen et al  (2013) [35] | Minimization algorithm | Computer-  generated code | Unclear | Unclear | Unclear | Low | Low | Low |
| O'Mahen et al  (2014) [36] | Minimization algorithm | Computer-  generated code | Unclear | Unclear | Unclear | Low | Low | Low |
| Shorey et al (2019) [37] | Computer software | Seal opaque envelopes | Low | Low | Low | Low | Low | Low |
